# Identification and Characterization of Salt- and Drought-Responsive *AQP* Family Genes in *Medicago* *sativa* L.

**DOI:** 10.3390/ijms23063342

**Published:** 2022-03-19

**Authors:** Yijing Luo, Lin Ma, Wenxuan Du, Su Yan, Zengyu Wang, Yongzhen Pang

**Affiliations:** 1 College of Grassland Science, Qingdao Agricultural University, Qingdao 266109, China; lyj199708@163.com (Y.L.); 17806284739@163.com (S.Y.); 2 Institute of Animal Science, Chinese Academy of Agricultural Sciences, Beijing 100093, China; malin@caas.cn (L.M.); n053727@163.com (W.D.)

**Keywords:** *Medicago sativa*, *AQP* genes, salt and drought stresses, aquaporin, phylogenetic analysis

## Abstract

Aquaporins (AQP) are distributed ubiquitously in plants, and they play important roles in multiple aspects of plant growth and development, as well as in plant resistance to various environmental stresses. In this study, 43 *MsAQP* genes were identified in the forage crop *Medicago sativa.* All the MsAQP proteins were clustered into four subfamilies based on sequence similarity and phylogenetic relationship, including 17 TIPs, 14 NIPs, 9 PIPs and 3 SIPs. Analyses of gene structure and conserved domains indicated that the majority of the deduced MsAQP proteins contained the signature transmembrane domains and the NPA motifs. Analyses on *cis*-acting elements in the promoter region of *MsAQP* genes revealed the presence of multiple and diverse stress-responsive and hormone-responsive *cis*-acting elements. In addition, by analyzing the available and comprehensive gene expression data of *M. truncatula*, we screened ten representative *MtAQP* genes that were responsive to NaCl or drought stress. By analyzing the sequence similarity and phylogenetic relationship, we finally identified the corresponding ten salt- or drought-responsive *AQP* genes in *M. sativa*, including three *MsTIPs*, three *MsPIPs* and four *MsNIPs*. The qPCRs showed that the relative expression levels of these ten selected *MsAQP* genes responded differently to NaCl or drought treatment in *M. sativa*. Gene expression patterns showed that most *MsAQP* genes were preferentially expressed in roots or in leaves, which may reflect their tissue-specific functions associated with development. Our results lay an important foundation for the future characterization of the functions of *MsAQP* genes, and provide candidate genes for stress resistance improvement through genetic breeding in *M. sativa*.

## 1. Introduction

Plants are exposed to various environmental stresses during their growth and development processes, including biotic and abiotic stresses, such as pathogens, salt, drought and cold [1,2]. Among these stresses, salt stress and drought stress are global environmental threats to the sustainable development of agriculture. It is estimated that at least one billion hm^2^ of saline-alkali land is distributed in more than 100 countries, and the arid and semi-arid regions account for about 30% of the total global area [3]. With the development of industrialization, agriculture and the economy, soil salinization has become more and more serious, which has not only discouraged the development of agriculture, but also led to serious ecological and environmental problems [4]. In the presence of excessive salt, osmotic stress and ionic toxicity are the two greatest challenges for plants, which lead to serious oxidative stress and nutritional disorders [2,4]. Drought could reduce plant growth, yield and adversely affect plant stability by affecting several morphological traits [5,6]. Therefore, investigation into the adaptation mechanism of plants to salt and/or drought stresses for further improvement is essential to accelerate agricultural production.

Salt as well as drought tolerance are complex traits that are controlled by multiple genes and involve various biochemical and physiology mechanisms [4,5]. Under either salt or drought stress, water transport driven by hydraulic and osmotic gradients plays an important role in plant adaption, besides in cell expansion, elongation, reproduction, absorption of mineral nutrients and phloem load [7]. In addition, the normal growth and development of plants are strongly dependent on the absorption of water by roots from the soil and the regulation of water movement in the plant. The diffusion of water across cell membranes is precisely regulated by water channels composed of aquaporins (AQPs), which are small (23–34 kDa) intact membrane proteins belonging to the major intrinsic protein (MIP) family [8,9].

AQP family proteins were ubiquitously distributed in the higher plants, in particular the model plants *Arabidopsis thaliana*, *Zea mays* and *Oryza sativa* [10,11,12]. These AQP proteins may transport water, small carbohydrates (such as glycerol), urea, NH_3_ and CO_2_ through energy-independent ion transport mechanisms [13,14]. So far, five subclasses of *AQPs* have been recognized from sequence comparisons and subcellular localization in plants, including: tonoplast intrinsic proteins (TIPs), plasma membrane intrinsic proteins (PIPs), nodulin 26-like intrinsic proteins (NIPs), small and basic intrinsic proteins (SIPs) and X intrinsic proteins (XIPs) [7,10,12,15]. Among them, the TIPs are predominately localized within the tonoplast membrane of the vacuoles of most plant cells [16,17]. The PIP proteins can be divided into PIP1 and PIP2, two distinct subcategories based on sequence comparisons and transport activity [10,12,18]. In comparison, the PIP1 subclass in general has a lower rate of facilitated water transport when compared to the PIP2 subclass. NIPs take their name from their subfamily patriarch, nodulin 26, which is found in symbiosomes of nitrogen-fixing nodules of soybean, and it is a major symbiosome membrane component and functions as an aquaglyceroporin [19]. NIPs’ subclass showed many characteristics of nodulin 26 and they may play broader roles in water homeostasis in non-nodulated plant tissues [19]. The SIPs’ subclass shows the most sequence divergence from the plant AQPs of other subclasses, in particular in the NPA box and the surrounding loop region [7,10,11]. It is possible that the transport selectivity and rate of these SIPs differ from the AQPs of other subclasses. The transport properties of the SIPs are not yet known, but the high amount of sequence divergence argues that they may have evolved separately from an ancestral gene [10,11]. XIPs are reported in some plant species, such as the tea plant and apricot [20,21], but not in the model plants such as *A. thaliana* or rice [11,12], and their functions are not very clear so far.

*AQP* are a large gene family in plants, and at least 35 different *AQP* genes were identified in *A. thaliana,* 31 in *Zea mays* and 33 in *Oryza sativa* [10,11,12], and the functions of many *AQP* genes have been characterized in different plant species, including *Camellia sinensis*, *Prunus armeniaca*, *Brassica rapa*, *Nicotiana tabacum*, *Fragaria ananassa*, and *Triticum turgidum* [20,21,22,23,24,25]. These AQP proteins were assumed to participate in different physiological processes, including the restriction of low pH-regulated rootlet water uptake [26], water use efficiency, hydraulic conductivity and photosynthesis [27]. Under favorable growth conditions, *AtPIP1b* over-expression significantly increased the growth rate, transpiration rate, stomatal density and photosynthetic efficiency in transgenic tobacco [28]. In particular, many *AQP* genes were reported to be responsive to abiotic stress during different developing stages [29,30], for example, the expression levels of seven and seventeen *AQP* genes from *Brassica napus* were responsive, respectively, to drought and salt stress during seed germination as well as in young seedlings [31]. The expression of *TaAQP7* and *TaAQP8* genes from wheat enhanced drought or salt tolerance in tobacco, respectively [32,33]. The over-expression of *T. turgidum TdPIP1;1* or *TdPIP2;1* in tobacco conferred strong tolerance towards salinity and osmotic stress [25]. The over-expression of the tobacco *Aquaporin1* gene in the tobacco plant improved water use efficiency, hydraulic conductivity and yield under salt stress [27]. All these results demonstrated that the *AQP* genes were responsive to abiotic stresses and they could be ectopically explored to improve plant resistance by over-expression.

Alfalfa (*Medicago sativa* L.) is a perennial legume forage crop, which is one of the most widely cultivated forage crops in the world, and it is also well-known as the “king of forages”, due to its rich content in protein, vitamins and other nutrients. With the continuous development of animal husbandry, the requirement for high-yield alfalfa is becoming higher and higher, especially under stressed conditions [34,35,36,37]. The growth and development of *M. sativa* were constrained by multiple environmental stress factors, such as drought, salt and extreme temperatures [6,35,36,37]. Alfalfa with strong salt and/or drought tolerance can provide high-quality protein feeds, which can smooth the way for the development of farming and animal husbandry in a saline-alkali or arid region [34]. Therefore, it is essential to investigate the salt and/or drought stress responsive mechanism and screen new genes for genetic breeding of *M. sativa*. So far, only one *AQP* gene *MsPIP2;2* from *M. sativa* was cloned, and its over-expression in *Arabidopsis* increased the seed germination rate, seedling root length, survival rate, proline content and antioxidant defense activity, but decreased the cell membrane damage and reactive oxygen species’ (ROS) accumulation under salt stress [38]. Apart from this single *MsAQP* gene, no other *AQP* family genes were cloned, nor was the entire gene family systematically investigated in *M. sativa*. Therefore, in the present study, efforts were taken to identify all putative *AQP* genes from *M. sativa*, along with their orthologs from *M. truncatula,* a model legume and a close relative of *M. sativa*. In addition, to provide comprehensive information on *MsAQP* genes, we also screened *MsAQP* genes that were highly responsive to salt or drought stress. These candidate genes could be applied in gene editing or genetic breeding for the improvement of alfalfa resistance against salt/drought stresses in the near future.

## 2. Results

### 2.1. Identification of AQP Family Genes in the Medicago Genome

Based on a homology search and PFAM analysis, a total of 88 *AQP* genes were identified in *M. sativa* (43) and *M. truncatula* (45) (Table 1 and Table S1). Their nucleotide and protein sequences were obtained from the *Medicago* genome database and used for further analyses. The physicochemical properties of the deduced AQP proteins are listed in Table 1 for *M. sativa* and Table S1 for *M. truncatula*, including the TIGR locus, homologous gene, molecular weights, isoelectric points and putative subcellular localization. The encoded protein length of the *MsAQP* genes range from 131 to 810 aa, with their molecular weight (MW) from 14.02 to 87.45 kDa and the isoelectric points (pI) from 4.08 to 9.78 (Table 1). It was predicted that the subcellular locations of most MsAQP proteins were in the cell membrane (Table 1). In addition, the corresponding *MsAQP* homologous genes from *M. truncatula* are also listed in Table 1, and the detailed information on those of the *MtAQPs* are shown in Table S1.

### 2.2. Multiple Sequence Alignment, Phylogenetic Analysis and Classification of AQPs in Medicago

**Figure 1:**
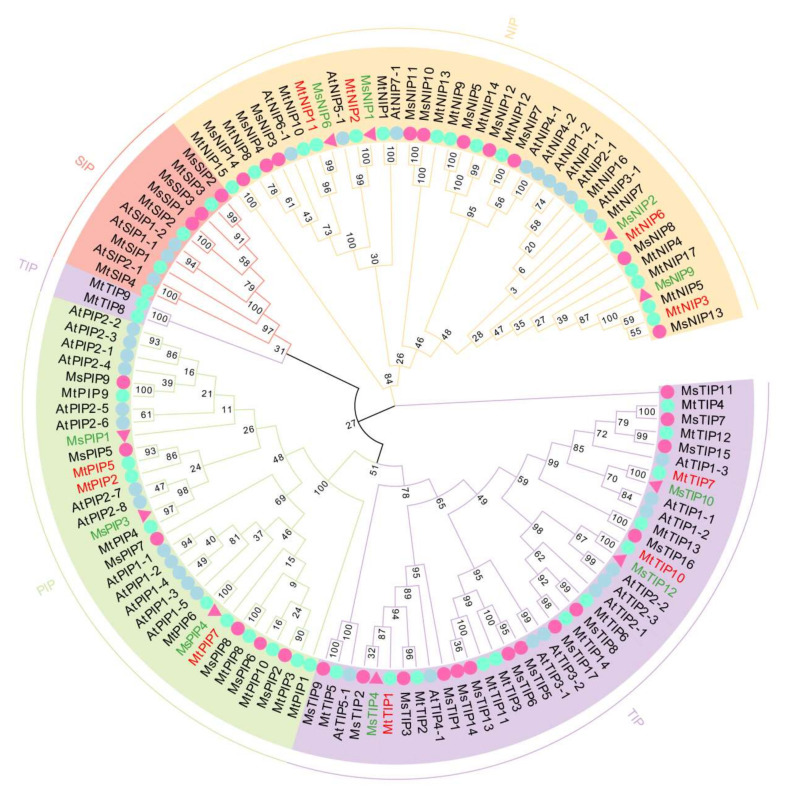
The phylogenetic analysis of AQP proteins from *M. sativa*, *M. truncatula* and *Arabidopsis*. The deduced full-length protein sequences of AQPs were constructed by using MEGA7 software based on the Maximum likelihood (ML) method, with bootstrap value of 1000 replicates. Members of four subfamilies of TIPs, PIPs, NIPs and SIPs are highlighted with purple, green, yellow and red backgrounds, respectively. The pink circles indicate AQP proteins from *M. sativa;* the cyan circles indicate AQP proteins from *M. truncatula;* and the light-blue circles indicate AQP proteins from *Arabidopsis*. The AQP proteins from *M. truncatula* are highlighted in red; while those from *M. sativa* are highlighted in green.

To investigate the evolutionary relationships of AQP from *M. sativa* and *M. truncatula*, a phylogenetic tree was constructed with the full amino acid sequences of all the AQP family proteins from *M. sativa* and *M. truncatula*, along with 35 AQP proteins from *A. thaliana* (Figure 1 and Table S2). It was shown that all AQP proteins could be classified into four distinct groups based on their sequence similarity, whether from *M. sativa*, *M. truncatula* or from *A. thaliana*: plasma membrane intrinsic proteins (PIPs, in green color), tonoplast intrinsic proteins (TIPs, in purple color), noduLin 26-like intrinsic proteins (NIPs, in yellow color) and small basic intrinsic proteins (SIPs, in red color), respectively (Figure 1).

Most of the AQP in *M. sativa* and *M. truncatula* were grouped with their homologs from *Arabidopsis* (Figure 1). In detail, 17 TIPs, 14 NIPs, 9 PIPs and 3 SIPs from *M. sativa*, and 10 PIPs, 14 TIPs, 17 NIPs and 4 SIPs from *M. truncatula* were grouped to the corresponding groups with those of *Arabidopsis*, respectively (Figure 1, Table S2). The largest cluster was for TIPs, with 17 members from *M. sativa* and 17 NIPs from *M. truncatula*, and the smallest cluster was SIPs with only three members from *M. sativa* (Figure 1). The phylogenetic clustering pattern of *AQPs* in *M. sativa* is consistent with that of *Arabidopsis*, showing a tendency to cluster with members of the same subfamily (Figure 1).

### 2.3. Chromosome Localization and Collinearity Analysis of AQP Genes in Medicago

**Figure 2:**
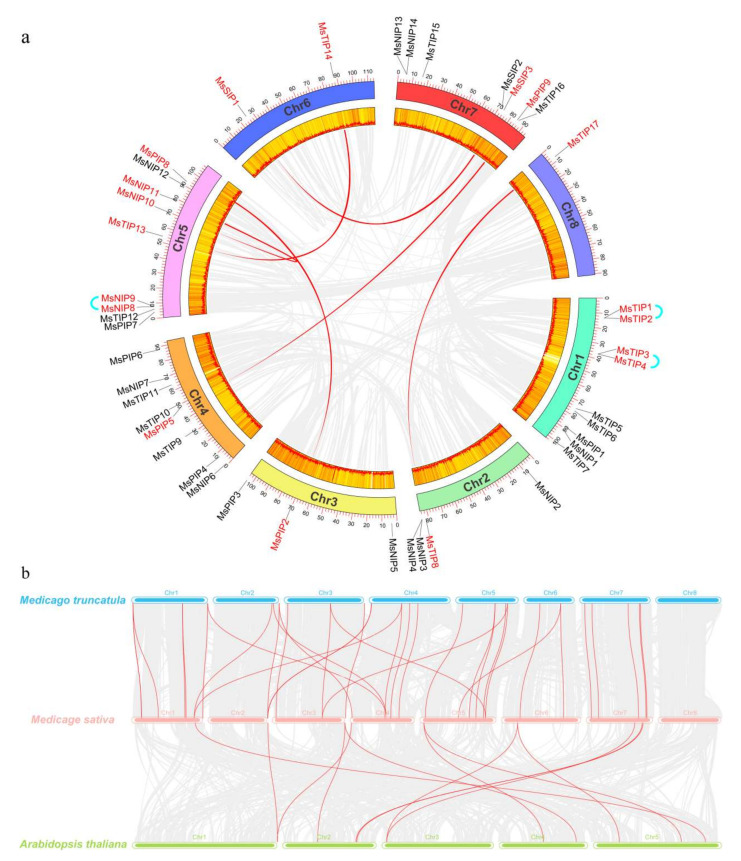
Chromosomal distribution of *AQP* family genes in *M*. *sativa*. (**a**) The chromosomal location and inter-chromosomal relationships of *AQP* genes on the eight chromosomes of *M*. *sativa*. The segmentally duplicated genes are in red and connected by red lines. The tandem duplicated genes are in red and connected by cyan lines; (**b**) Synteny analysis of *AQP* genes among *M. sativa*, *Arabidopsis*, and *M. truncatula*. Gray lines in the background indicate the collinear blocks within *M. sativa*, and *M. truncatula*/*Arabidopsis*, and the orange lines highlight the syntenic *AQP* gene pairs.

Overall, 43 *MsAQP* genes were randomly distributed in eight chromosomes of *M.* *sativa* (Figure 2a). Nine *MsAQP* genes were located in chromosome 1 and 5, eight genes in chromosome 4, seven in chromosome 7, four in chromosome 2, three in chromosome 3, two in chromosome 6 and only one gene in chromosome 8 (Figure 2a). Among them, chromosomes 1 and 5 had the most *AQP* genes (9 *TIP* genes), and chromosome 8 had the least *AQP* genes (only *TIP17* gene), all the other chromosomes have two to eight *AQP* genes (Figure 2a).

In addition, both tandem and segmental duplication occurred in the generation of the *MsAQP* family gene during the evolution. Three tandem duplicates were identified for *MsAQP* genes: *MsNIP8*/*MsNIP9*, *MsTIP1*/*MsTIP2* and *MsTIP3*/*MsTIP4*. Six segmental duplication events were also identified, including *MsTIP8*/*MsTIP17, MsPIP2*/*MsPIP8*, *MsPIP5*/*MsPIP9*, *MsNIP10*/*MsNIP11*, *MsTIP13*/*MsTIP14* and *MsSIP1*/*MsSIP3* (Figure 2a, Additional file S1). All these results indicated that both tandem duplication and segmental duplication events played an important role in the evolution of *AQP* genes in *M. sativa*.

Furthermore, a comparative syntenic map with *M. sativa*, *M*. *truncatula* and *Arabidopsis* was constructed to illustrate the evolution relationship of the *AQP* gene family (Figure 2b). In total, 12 and 33 orthologous pairs were found between *M. sativa* and *Arabidopsis*, and between *M. sativa* and *M. truncatula*, respectively (Figure 2b, Additional file S1). Taken together, these results indicated that the *AQP* genes of *M. sativa* may have undergone a strong purification selection pressure during evolution.

### 2.4. Analyses of Conserved Motif and Gene Structure of AQP Genes in M. sativa

AQP proteins usually have an overall membrane topology consisting of six transmembrane spanning alpha helical domains (TM1-TM6) connected by five loop regions [39]. The NPA regions are the signature motifs for AQP family members [39], which are symmetrically oriented sequences of asparagine-proline-alanine (NPA, Figure S1). Six transmembrane domains were detected in twenty-eight members, and they showed relatively higher sequence similarity than in the other region (Figure S1). However, the remaining 15 MsAQP proteins lacked at least one of the six transmembrane domains (Figure S1). Moreover, two NPA domains were detected in 31 MsAQP proteins, while the other 12 MsAQP proteins (MsNIP2, MsNIP3, MsNIP4, MsNIP10, MsNIP11, MsTIP1, MsTIP3, MsTIP5, MsTIP11, MsTIP13, MsPIP6 and MsPIP7) possessed only one of the NPA domains. The lack of at least one of the transmembrane domains or NPA domains may lead to the improper topological structure for a normal function of AQP proteins.

**Figure 3:**
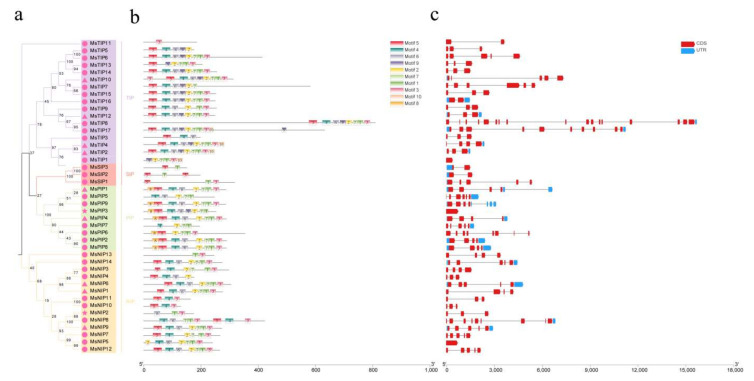
Analyses of the phylogenetic relationships, motifs and gene structure of *AQP* genes from *M. sativa*. Phylogenetic relationships (**a**); location of motifs (**b**); and gene structure of *MsAQP* genes (**c**). In (**c**), blue boxes indicate 5′- or 3′- untranslated regions, red boxes indicate exons, and black lines indicate introns.

Analysis on motifs showed that the MsAQPs protein members of the same subfamily shared a high similarity in the conserved motifs (Figure 3a). Multiple motifs, including motifs 5, 4, 6 and 9, and motifs 2, 7, 1 and 3 in the TIPs proteins, motifs 8, 5, 6, 4 and motifs 2, 7, 1, 3 in the PIPs proteins, motifs 5, 4, 6 and motifs 2, 7, 1 and 3 in the NIPs proteins, motif 5 and motif 1 in the SIPs proteins were the conserved motifs, which, respectively, contained the two conserved NPA domains (Figure 3b). Motifs 1 and motif 3 were common to all subfamilies, TIPs, PIPs and NIPs contain conserved motifs 5, 4 and 6, differing by one additional motif 9 in TIPs and motif 8 in PIPs (Figure 3b), which can be used to distinguish these subfamilies.

The gene structure of *MsAQPs* was also analyzed, and it showed that each *MsAQP* has multiple introns. Twelve *AQP* members have introns of more than five, including *MsTIP7*, *8*, *17*, *MsPIP1*, *5*, *6*, *7*, *9*, *MsNI6*, *8*, *9*, *14*, while the other seventeen genes have introns of less than two (Figure 3c). In particular, three *MsAQP* genes do not have any intron (*MsTIP1*, *MsPIP3* and *MsNIP5*). The structural differences in the *AQPs* may allow them to function differently. However, it has to be noted that the structure of many of the *MsAQP* genes are not complete, which may be due to the incompleteness of the genome sequence assembly of *M. sativa*.

### 2.5. Analyses of Cis-Acting Elements of MsAQP Genes

**Figure 4:**
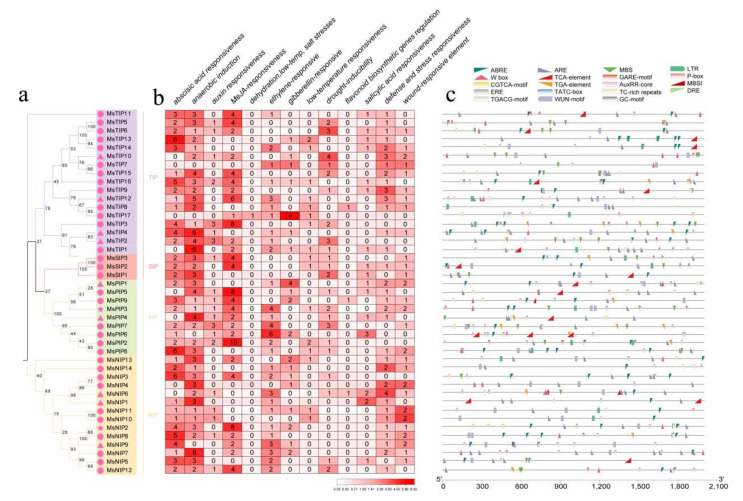
Analyses of *cis*-acting elements in the upstream promoter region of *AQPs* in *M. sativa*. (**a**) The phylogenetic analysis of the deduced AQP proteins of *M. sativa;* (**b**) The colors and numbers on the grid indicated the numbers of different *cis*-acting elements in these *MsAQP* genes, which is presented in the form of heatmap; (**c**) Colored blocks represented different types of *cis*-acting elements and their relative location in the promoter region of each *MsAQP* gene.

The upstream promoter regions of 2000 bp for all the *AQP* genes from *M. sativa* were analyzed. Those hormone-responsive and abiotic stress-related *cis*-acting elements were identified, including abscisic acid responsiveness (ABRE), anaerobic induction (ARE, GC-motif), auxin responsiveness (AuxRR-core, TGA-element), MeJA-responsiveness (CGTCA-motif, TGACG-motif), dehydration, low-temp and salt stresses (DRE), ethylene-responsiveness (ERE), gibberellin-responsiveness (GARE-motif, P-box, TATC-box), low-temperature responsiveness (LTR), drought-inducibility (MBS), flavonoid biosynthetic genes’ regulation (MBSI), salicylic acid responsiveness (TCA-element), defense and stress responsiveness (TC-rich repeats, W box) and wound-responsive element (WUN-motif) (Figure 4 and Additional file S2).

It was shown that the promoters of *MsAQP* genes contained various *cis*-acting elements with different numbers (Figure 4a). We found that the *cis*-acting elements related to ABA stress responsiveness, anaerobic stress and MeJA stress are widely distributed in the *MsAQP* family genes. In addition, most *MsAQP* genes contain the *cis*-acting elements related to defense and environmental stress, drought stress and salt-alkali stress responsiveness (Figure 4b). DRE related to dehydration, low-temp and salt stresses, is only presented in *MsTIP17*, and MBSI that is involved in flavonoid biosynthetic genes regulation is presented in three *MsAQP* members (*MsTIP8*, *MsPIP9* and *MsNIP6*, Figure 4b). Among all *MsAQP* members, *MsTIP13*, *MsTIP16*, *MsPIP8*, *MsNIP3* and *MsNIP8* have at least five ABRE elements, *MsTIP12*, *MsTIP4*, *MsTIP1* and *MsNIP7* have at least five ARE repeats (Figure 4a,b).

In addition, MeJA-responsiveness elements (CGTCA-motif, TGACG-motif) were widely distributed in all the *MsAQP* promoter regions, with *MsPIP2* having the most number of 10 (Figure 4a,b). *MsTIP1*, *6*, *9*, *11*, *13*, *14*, *16*, *MsPIP1*, *4*, *5*, *6*, *MsNIP1*, *5*, *6* and *MsSIP1,* two contain at least one TCA-element related to salicylic acid responsiveness (Figure 4a,b). Moreover, *MsTIP4*, *5*, *6*, *7*, *9*, *10*, *11*, *12*, *15*, *MsPIP1*, *3*, *5*, *8*, *9*, *MsNIP2*, *4*, *6*, *7*, *8*, *9*, *12*, *13* and *MsSIP2* contains W box that is related to defense and stress responsiveness (Figure 4a,b). In particular, *MsTIP16*, *MsTIP12*, *MsPIP2* and *MsNIP2* have at least 20 different types of *cis*-acting elements (Figure 4a,b). The presence of various *cis*-acting elements in *MsAQP* genes indicated that they might be involved in various stress responses and hormone pathways, in particular those that have various types of *cis*-acting elements with a large number.

### 2.6. Analyses of Expression Profiles of MtAQPs for the Screening of Candidate MsAQP Genes

Currently, no comprehensive transcriptome data were available for *M. sativa* to further analyze the expression profiles of *MsAQP* genes. However, *M. truncatula* has a number of GeneChip^®^ data for screening of the candidate genes. More importantly, *M. truncatula* is the closest relative to *M. sativa,* both in phylogenetic relationship and in gene sequences. Theoretically, their homologs may share similar expression profiles. Therefore, in order to further analyze the function of *MsAQPs*, the strategy we used was to firstly analyze the expressions’ profiles of the homology *MtAQP* genes under salt or drought-treatment for the screening of stress-responsive *MtAQP*, and then verify their corresponding homology genes in *M. sativa* (Table 1). In this way, we obtained the expression profiles of 29 *MtAQP* with available comprehensive GeneChip^®^ data for *M. truncatula*.

**Figure 5:**
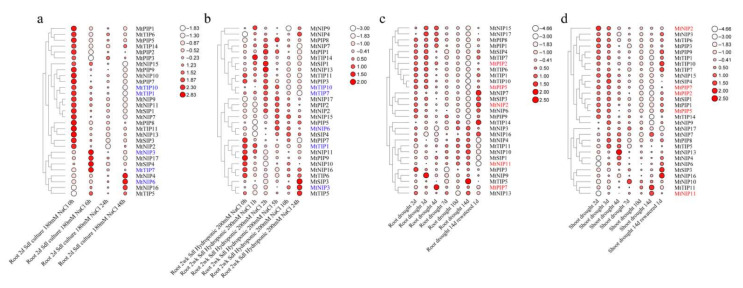
Expression profiles of *MtAQP* genes under different treatments at different time points were retrieved from the *M. truncatula* gene expression atlas with multiple GeneChip^®^ data. The color of circles from white to red indicates the expression level from low to high after normalization. (**a**) Expression profiles of *MtAQP* genes in roots under 180 mM NaCl treatment at 0, 6, 24 and 48 h; (**b**) Expression profiles of *MtAQP* genes in roots under 200 mM NaCl treatment at 0, 1, 2, 5, 10 and 24 h; (**c**) Expression profiles of *MtAQP* genes in roots under drought treatment at 2, 3, 4, 7, 10, 14 d and rewater 1d after 14 d drought treatment; (**d**) Expression profiles of *MtAQP* genes in shoots under drought treatment at 2, 3, 4, 7, 10, 14 d and rewater 1 d after 14 d drought treatment.

With an emphasis on the screening of salt-responsive *MsAQP* genes, we analyzed their gene expression profile in the roots of 2-day-old seedlings that were treated with 180 mM NaCl at 0, 6, 24, 48 h (Figure 5a), and the roots of 2-week-old seedlings that were treated with 200 mM NaCl at 0, 1, 2, 5, 10, 24 h, respectively, from the online GeneChip^®^ database (Figure 5b). The expression levels of most *MtAQP* genes were decreased over time under the 180 mM NaCl treatment, while only three genes (*MtNIP4*, *MtNIP6* and *MtNIP16*) increased significantly at 48 h (Figure 5a), and four genes increased rapidly at 6 h (*MtNIP3*, *MtNIP17*, *MtSIP4* and *MtTIP7*), but decreased at 24 h (Figure 5a). Under 200 mM NaCl treatment, the expression levels of most of the *MtAQP* members in roots increased at different time points, and then decreased afterwards (Figure 5b). Moreover, the expression levels of seven genes (*MtPIP7*, *MtTIP1*, *MtNIP11*, *MtPIP9*, *MtNIP10*, *MtNIP16* and *MtTIP6*) decreased continuously (Figure 5b). In addition, the expression levels of three genes (*MtSIP3*, *MtNIP3* and *MtTIP5*) increased gradually and reached the highest level at 24 h (Figure 5b).

Apart from the salt-stress responsive *MtAQPs*, we also investigated the expression changes of *MtAQP* gene members that related to drought stress. We explored the expression profile of *MtAQP* genes in roots and shoots that were under drought treatment for 2 d, 3 d, 4 d, 7 d, 10 d, 14 d and rewatered 1 d after 14-day of drought treatment. As was shown in Figure 5c–d, it was clear that the response of *MtAQPs* were different from each other under drought treatment. In details, the expression levels of five genes (*MtNIP7*, *MtSIP3*, *MtNIP2*, *MtNIP16* and *MtTIP14*) reached the highest level at 1 d rewater after 14 d drought stress (Figure 5c). In addition, the expression levels of five genes (*MtNIP4*, *MtTIP11*, *MtNIP10*, *MtSIP1* and *MtNIP11*) increased gradually from 2 d to 14 d (Figure 5c). In the shoots, the expression levels of most *MtAQP* members were decreased from 2 d to 14 d under drought treatment (Figure 5d). However, the expression levels of two genes (*MtTIP11* and *MtNIP11*) reached the highest level at 14 d under drought treatment (Figure 5d). In addition, the expression levels of 4 genes (*MtNIP6*, *MtNIP4*, *MtSIP3* and *MtNIP16*) increased gradually from 2 d to 14 d (Figure 5d).

Among all *MtAQP* genes, five of them were found to be highly responsive to salt stress with a distinct expression pattern (*MtTIP1*, *MtTIP7*, *MtTIP10*, *MtNIP3* and *MtNIP6*, in blue in Figure 5a,b), while another five (*MtNIP2*, *MtNIP11*, *MtPIP1*, *MtPIP7* and *MtPIP9*, in red in Figure 5c,d) for drought stress (Figure 5 and Additional file S3), which were typical representatives of *MtAQP* genes. Due to the close relationship of *M. sativa* to *M. truncatula*, as well as the close phylogenetic relationship of *AQP* genes between *M. sativa* and *M. truncatula* (Figure 1), we screened the corresponding 10 homology gene pairs: including *MtTIP1/MsTIP4*, *MtTIP7/MsTIP10*, *MtTIP10/MsTIP12*, *MtNIP3/MsNIP9*, *MtNIP2/MsNIP1*, *MtNIP6/MsNIP2*, *MtNIP11/MsNIP6*, *MtPIP5/MsPIP1*, *MtPIP7/MsPIP4* and *MtPIP2/MsPIP3* (Figure 1). By using this strategy, the 10 candidate *AQP* genes from *M. sativa* that responded to salt stress or drought stress were preliminarily selected for further analyses as representatives.

### 2.7. Validation of Expression Profile of Stress-Responsive MsAQP Genes by qPCR Analysis

**Figure 6:**
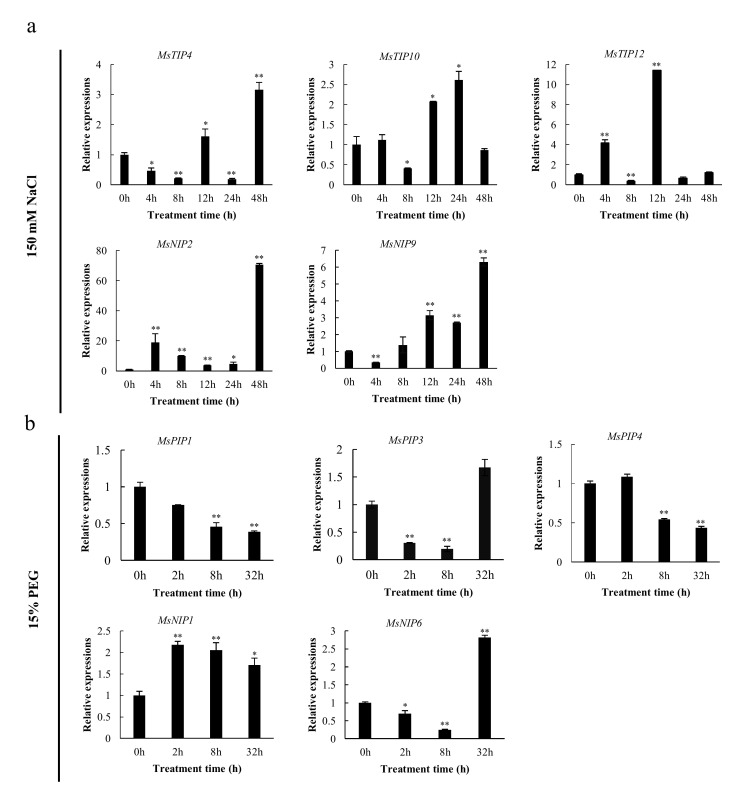
Determination of gene expression levels for 10 *MsAQP* genes under stresses by using qPCR. Data are the average of three independent biological samples ± SE and vertical bars indicate standard deviation. (**a**) The value of X axis represents treatment period with 150 mM NaCl at 0, 4, 8, 12, 24 and 48 h, respectively; the value of Y axis represents relative expressions of each gene; (**b**) The value of X axis represents treatment period with 15% PEG at 0, 2, 8 and 32 h, respectively; the value of Y axis represents the relative expression of each gene. Data are presented as mean ± SD, Student’s *t*-test (*n* = 3, * *p* <0.05, ** *p* <0.01).

The relative expression of these selected representative genes was further determined under 150 mM NaCl and 15% polyethylene glycol (PEG) stress treatments at different time points, respectively (Figure 6). Under 150 mM NaCl treatment, the relative expression levels of these five genes evidently increased at different time points with a different fold change. For *MsTIP4*, its relative expression level increased at 12 h after a slight decrease at 4 h and 8 h, and it reached the peak level at 48 h by more than 3-fold (Figure 6a, upper panel). For *MsTIP10*, its relative expression level was relatively higher at 12 h and 24 h with 2–2.5-fold changes (Figure 6a, upper panel). The relative expression level of *MsTIP12* increased more than 4-fold at 4 h followed by a decrease, and then reached the peak at 12 h by more than a 10-fold change (Figure 6a, upper panel). For *MsNIP2*, its relative expression level increased from 4–48 h, and reached the highest level at 48 h (Figure 6a, lower panel). The relative expression of *MsNIP9* decreased slightly at 4 h and then increased relatively stable from 8 h to 48 h with the highest fold change of more than 6 times at 48 h (Figure 6a, lower panel).

Under PEG treatment, the relative expression level of *MsPIP1* decreased from 2 h to 32 h (Figure 6b), and that of *MsPIP3* decreased at 2 h and 8 h, but increased slightly at 32 h (Figure 6b). For *MsPIP4*, its relative expression level decreased at 8 h and 32 h (Figure 6b). For the two *MsNIP* genes, the relative expression level of *MsNIP1* was significantly increased by 2–2.5 fold at 2h, 8 h and 32 h (Figure 6b), while *MsNIP6* showed a similar expression profile as MsPIP3, increased at 2 h and 8 h, and increased at 32 h. Overall, the fold changes of all selected drought-responsive genes did not change as much as the salt-responsive genes.

### 2.8. Spatial Expression Profiling of MsAQP

**Figure 7:**
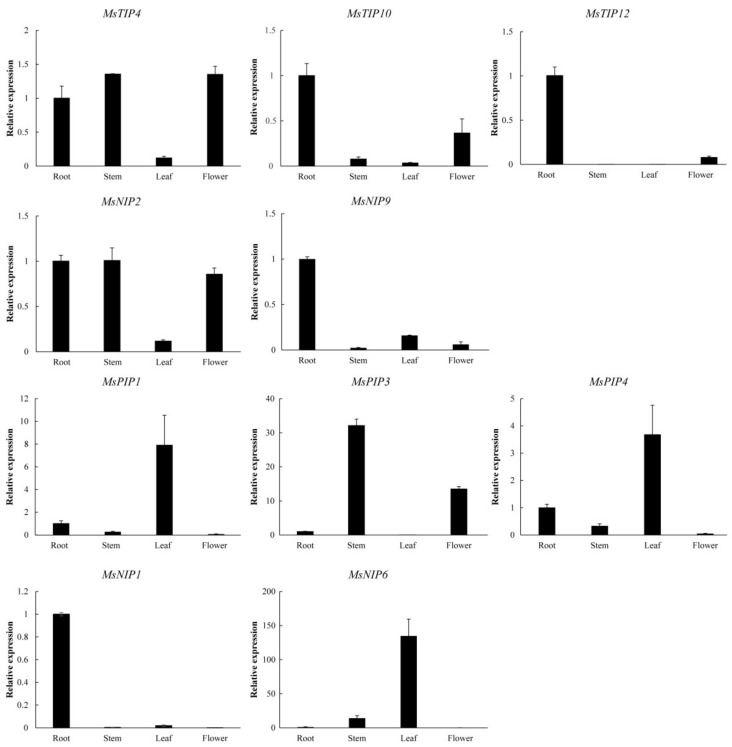
Determination of relative expression levels for 10 *MsAQP* genes in different tissues in *M. sativa* by using qPCR. Data are the average of three independent biological samples ± SE and vertical bars indicate standard deviation. The value of the X axis represents root, stem, leaf, flower tissues. The expression level in root for each sample were set as value of 1.

The expression levels of the above-mentioned 10 *MsAQP* genes were further investigated in the roots, stems, leaves and flowers (Figure 7). It was shown that the relative expression level of these 10 *MsAQP* genes exhibited distinct expression profiles. It was clear that the three *MsTIP* genes (*MsTIP4*, *MsTIP10*, and *MsTIP12*) responsive to NaCl treatment were highly expressed in the roots, with *MsTIP4* also highly expressed in stems and flowers, and it was the same for the two *MsNIPs* (*MsNIP2* and *MsNIP9*) (Figure 7, first and second panel). As for the five drought-responsive *MsAQP* genes, two of them were preferentially expressed in the leaves (*MsPIP1* and *MsPIP4*), and one of them was preferentially expressed in the stems (*MsPIP3*) (Figure 7, third panels). In addition, *MsNIP1* and *MsNIP6* showed the highest expression levels in roots and leaves, respectively (Figure 7, lower panel). These data taken together indicated that these *MsAQP* genes participate in the growth and development of specific tissues in *M. sativa*, functioning alone or in combination in the same tissues.

## 3. Discussion

Many studies on alfalfa resistance to abiotic stress have been focused on various types of genes, in particular transcription factors, including *MYB*, *ERF*, *HLH* etc. [40,41,42,43]. These regulatory genes regulated multiple pathways or genes, but investigation on the mechanism of direct water regulation by the individual *AQP* gene was rare in alfalfa. *AQPs* play an important role in maintaining the cell water dynamic balance, which plays a critical role in plant growth and development under drought or salt tolerances, due to the osmotic stresses and ion toxicity [9,44]. Many plant *AQP* genes involved in drought and salt stress have been identified, but only one *AQP* gene was characterized in *M. sativa* so far. In a previous study, a PIP-type *AQP* gene *MsPIP2;2* was identified by RACE techniques [38]. The closest homolog of *MsPIP2;2* was *MtPIP5* in *M. truncatula*, which was inducible by 200 mM NaCl in two-week-old seedlings in our study. This phenomenon was consistent with the results that the expression level of *MsPIP2;2* was increased by 150 mM NaCl treatment after a short time treatment at 2 h and 4 h in *M. sativa* [38]. The expression level of *MsPIP2;2* did not show a significant change as a result of drought treatment [38], which did not agree entirely with our results that its expression (as *MsPIP1* in Figure 6b) was slightly reduced by PEG treatment, although the fold change was not very high.

In this study, we identified 43 *MsAQP* in the genome of *M. sativa* and 45 *MtAQP* genes in *M. truncatula* (Table 1 and Table S2). Compared with around 35 members in *A. thaliana*, 31 in maize, and 33 in rice [10,11,12], the number of *AQP* family members in *Medicago* was expanded to 43 during evolution. An increase in gene numbers could be due to gene duplication events. When compared with another large gene family with similar members, as in our previous study for *CBL* and *CIPK* genes that have only one segmental duplicate, and three to four tandem duplicates [45], the *MsAQP* gene family has more segmental duplicates (six) and less (three) tandem duplicates, indicating segmental duplicate played a more important and direct role than tandem duplicates in the evolution of the *AQP* gene family in *M. sativa.* This phenomenon of tandem and segmental repetition exists in other plant protein families and is the result of gene replication in the process of evolution. Further, in *Arabidopsis* [12], the PIP subfamily had the most AQP members with 13 members, but in *M. sativa*, the TIP subfamily had the most AQP members, with 17 members (Table 1), which may be the main reason for the expansion of the *AQP* gene family in *M. sativa*. It is interesting that XIPs were identified from some plants, such as the common bean, *nicotiana glauca*, tea plant, apricot [20,21,23,46], however, XIP was absent in *M. sativa* as in *Arabidopsis* and *Brassica rapa* [12,31], which indicated that XIPs might be unique for specific families of plant.

The amino acid composition and three-dimensional structure of aquaporins are very important for the function of aquaporin in transporting substrate molecules. Meanwhile, the loss or acquisition of the transmembrane domain may affect subcellular localization, amino acid sequence folding and the efficiency of water transporting [8,13,15,44]. However, based on the genome sequence of alfalfa, the length of MsAQP proteins was as short as 131 aa for MsNIP10, and as long as 810 aa for MsTIP8 (Table 1). In addition, we found that almost one third of total MsAQP proteins lost one or more of the conserved domains, at least one to three transmembrane domains or one NPA domain (Figure S1). By contrast, the length of the deduced AQP proteins ranged from 240 to 323 aa in *A. thaliana* [12], from 219 to 329 aa in apricots [21], 236 to 326 aa in tea plants [20]. Even in the close relative of *M. truncatula*, the length of the majority of MtAQPs fell into the range of 218–330 aa (Table S1), therefore, we suspected that the lack of partial gene sequences may be due to the incompleteness of the genome assembly. As a trial, we cloned the *MsNIP2* gene by PCR, as expected, we found its extract length was 272 aa after sequencing the coding genes (816 nt). Therefore, it could be reasonably predicted that the particular relative short or long MsAQP may be due to the incomplete assembly of genome sequencing, which could be improved by better assembly technique or by experimental confirmation.

Since the C-terminal of PIPs is shorter than other subfamily protein sequences [14], there were significant differences in subcellular localization among different subfamilies of aquaporins. PIPs, NIPs and some SIPs were located on the cell membrane, most of TIPs on the vacuole and some on the cell membrane [47] and some SIPs were located on the endoplasmic reticulum [48]. In the present study, it was predicted that the majority of the MsAQP proteins were localized in the cell membrane (Table 1), beside the vacuole, mitochondrion and endoplasmic reticulum, and whether they are localized in tonoplast, endoplasmic reticulum or mitochondrion needs further investigation in the near future. Nevertheless, the multiple and diverse localizations of MsAQP indicate their various functions in different cell compartments.

It has been reported that under abiotic stresses such as drought, cold, salt, mechanical injury, osmotic stress, heavy metal and flooding hypoxia, most *AQPs* expression levels were decreased, thus limiting water transporting in plants, maintaining water balance and increasing tolerance to stress factors [15]. In this study, ten *MsAQP* genes were found to be differently responsive to salt or drought stress (Figure 6). Among them, three *MsTIP* genes (*MsTIP4*, *MsTIP10* and *MsTIP12)* and two *MsNIP* genes (*MsNIP2* and *MsNIP9*) were inducible by NaCl treatment, and they responded differently, while three *MsPIP* genes (*MsPIP1*, *MsPIP3* and *MsPIP4*) and two *MsNIP* genes (*MsNIP1* and *MsNIP6*) were inducible under PEG treatment (Figure 6). Among these genes, at least three *MsPIP* genes out of all nine *MsPIP* genes were associated with drought treatment, which is consistent with many other studies showing that a high percentage of *PIP* genes were associated with drought or salt stress [38,49,50]. Among them, the *MsPIP1* gene was downregulated by PEG treatment, which was the same as several *AQP* genes that were downregulated under drought stress in *Nicotiana glanuca* [23]. This could be explained by the reason that these AQP likely function in limiting water loss while others maintain homeostasis in the cell. Nevertheless, the specific reasons and mechanisms of each individual gene need to be further studied.

The function of the *AQP* family is to transport water, glycerin and small molecules in the plant body, but their specific transporting mechanism is little known currently. It is known that different subfamilies of AQP aquaporins are regulated and expressed sequentially at different stages of plant growth and development, thereby completing the full life process of plants [51]. In our study, candidate *MsAQP* genes responsive to salt and drought stress were differently expressed in the main tissues of *M. sativa*. It was obvious that all salt-responsive *MsAQP* genes were more or less abundantly expressed in roots compared to other tested tissues (Figure 7). The results were consistent with the expression profiles of the corresponding *MtAQP* that were inducible at different level in the roots (Figure 5a,b). Our results also agree with other studies that the expression level of many *PIP* genes were upregulated in the root under salt stress, as in rice and maize [52,53]. While the drought-responsive *MsAQP* genes were highly expressed in the roots, stems, leaves and flowers (Figure 7), which was mainly because these homology genes were selected from the gene expression profile in both roots and shoots (Figure 5c,d). Meanwhile, it also indicated that drought treatment resulted in the expression of different types of *MsAQP* in many tissues of *M. sativa*, and they might function in combination against drought stress. The similar phenomena were also found in rice and maize that drought caused the upregulation of several *AQP* genes in roots and stems of rice [53].

It this study, we identified and analyzed all putative *AQP* genes in *M. sativa*, selected new candidate salt and drought responsive *MsAQP* genes, and these genes may have potential important functions in regulating water homeostasis under abiotic stress in *M. sativa*. Whether they have special mechanisms in stress tolerance and whether they could be utilized for genetic breeding is worthy of in-depth study.

## 4. Materials and Methods

### 4.1. Identification of AQP Genes in the Medicago Sativa and Medicago Truncatula Genome

The genome sequences and deduced protein sequences of *AQPs* were downloaded from the *M. sativa* genome website (https://figshare.com/articles/dataset/Medicago_sativa_genome_and_annotation_files/12623960, accessed on 2 June 2021) and the *M*. *truncatula* genome website (http://www.medicagogenome.org/, accessed on 9 June 2021). The Hidden Markov model (HMM) profiles of MIP (PF00230) were downloaded from the Pfam database (https://pfam.xfam.org/, accessed on 15 June 2021) and used as the query (*p* < 1 × 10^−5^) to search putative *AQP* proteins from the *M. sativa* and *M. truncatula* genome database. Moreover, all *AQP* protein sequences in *Arabidopsis* were downloaded from TAIR (https://www.arabidopsis.org/, accessed on 3 June 2021), which were applied as a query (*p* < 1 × 10^−5^) to blast search the protein sequences of *M. sativa* and *M. truncatula* in their genome database. All output candidate AQP protein sequences that may contain AQP domain based on HMMER and the BlastP results were confirmed by the presence of the AQP core sequences submitted to Pfam (https://pfam.xfam.org/, accessed on 15 June 2021), InterProScan (https://www.ebi.ac.uk/interpro/search/sequence-search, accessed on 15 June 2021), SMART (http://smart.embl-heidelberg.de/, accessed on 15 June 2021) program. and CDD (https://www.ncbi.nlm.nih.gov/Structure/bwrpsb/bwrpsb.cgi, accessed on 15 June 2021) to confirm the conserved AQP domain. In total, 43 candidate *MsAQP* and 45 candidate *MtAQP* genes were obtained and further assigned based on their locations on chromosomes (Table 1 and Table S2).

### 4.2. Sequence Analysis and Structural Characterization of AQP Genes in M. sativa

Sequence alignment analysis of *AQP* domain sequences were performed by jalview (http://www.jalview.org/Web_Installers/install.htm, accessed on 14 September 2021). All the sequences of *AQP* genes were submitted to the ExPASy website (http://web.expasy.org/protparam/, accessed on 2 June 2021) to the length of amino acids, the molecular weight and theoretical isoelectric points. Conserved motifs in AQP protein sequences were identified by the MEME program (MEME-Suite version 5.1.0, http://meme-suite.org/, accessed on 2 June 2021) [54], with default settings and the motif number was set at a value of 20. Subcellular locations of AQP proteins were predicted by using the online program Plant-mPLOC (http://www.csbio.sjtu.edu.cn/bioinf/plant-multi/, accessed on 3 June 2021). The predication and visualization of exon-intron positions and conserved motifs were performed through Visualize Gene Structure program by using the software of TBtools [55].

### 4.3. Phylogenetic Analysis and Classification of the AQP Genes in M. sativa

In total, one hundred and twenty-three deduced AQP protein sequences from *M. sativa*, *M. truncatula* and *Arabidopsis* were used to construct the phylogenetic tree with the Maximum likelihood (ML) method by using MEGA7 software [56]. The default parameters were used to construct the phylogenetic tree, with 1000 bootstrap replicates. The online program EvolView (https://evolgenius.info/evolview-v2/, accessed on 27 September 2021) was used to optimize the evolutionary tree.

### 4.4. Analyses of Collinearity and Chromosome Location of the AQP Genes

The multiple collinear scan toolkit (MCScanX) was used to analyze gene repetitive events with default parameters [57]. The chromosomal location of all *AQPs* of *M. sativa* was analyzed with Advanced Circos program by using the software TBtools [55], and all *MsAQP* genes were mapped to the eight *M. sativa* chromosomes based on their physical location from the *Medicago* genome database. Gene Location Visualize program in the software of TBtools [55] were used to analyze the intraspecific synteny relationship among *M*. *sativa*, *M. truncatula* and *Arabidopsis*.

### 4.5. Analysis of Cis-Acting Element of the MsAQP Genes

The *cis*-acting elements in the upstream 2000 bp promoter sequences of the *MsAQP* genes were submitted to online program PlantCARE (http://bioinformatics.psb.ugent.be/webtools/plantcare/html/, accessed on 15 June 2021) [58]. Then the information was further analyzed and shown as a heatmap with the Heatmap Illustrator program in the software of TBtools [55].

### 4.6. Analysis of the Expression Profile of MtAQP Genes with Microarray Data

We downloaded all the microarray data from the *M. truncatula* Gene Expression Atlas (https://Mtgea.noble.org/v3/, accessed on 18 July 2021), and then analyzed the expression level of 29 available *MtAQP* genes. The selected microarray data contained the shoots and roots samples subjected to salt stress and drought stress treatment. Two-day-old seedlings were used for the 180 mM NaCl treatment, and the root samples were collected for qPCR, at time points of 0, 6, 24 and 48 h. Meanwhile, two-week-old seedlings were also used for the 200 mM NaCl treatment under hydroponic culture, and the root samples were collected at time points of 0, 1, 2, 5, 10 and 24 h. Plants were used for drought treatment at time points of 2, 3, 4, 7, 10, 14 d and 1 d rewater after 14 d drought, then the root and shoot samples were, respectively, collected for qPCR analyses. The relative expression profiles were analyzed and visualized as heatmap by using the HeatMap Illustrator program in the software of TBtools [55].

### 4.7. Plant Materials and Treatments

The *M. sativa* (cv. Zhongmu No.1) plants used in this study, and the leaves of 4-week-old *M. sativa* seedlings under hydroponic condition, were collected for RNA extraction and used for further qPCR analysis. To investigate the expression level of candidate *MsAQP* genes under different stresses, plants were grown in a growth chamber at 24 °C with 16 h light/8 h dark photoperiod for subsequent analysis. The 150 mM NaCl were used for salt stress treatment at time points of 0, 4, 8, 12, 24 and 48 h. The 15% PEG was used for drought stress treatment at time points of 0, 2, 8 and 32 h.

For the spatial expression analyses of *MsAQP* genes, the seedlings were grown in the greenhouse under 16/8 h light/dark regime at 25 °C. Roots, stems, leaves and flowers were taken at the initial flowering stage from three individual plants with similar growth condition.

### 4.8. Analysis of the AQP Gene Expression in M. sativa by qPCR

Total RNAs were isolated by using the Eastep Super total RNA Extraction kit (Promega, Shanghai, China) according to the manufacturer’s instructions. First-strand cDNA synthesis was carried out by using the Trans Script One-Step gDNA Removal and cDNA Synthesis SuperMix (TransGen Biotech, Beijing, China) according to the manufacturer’s instructions. The quantitative real-time PCR (qPCR) procedures and methods were performed by using the 2×RealStar Green Fast Mixture (GeneStar, Shanghai, China) on the ABI 7500 real-time Detection System (Applied Biosystems, USA). The detailed procedure for qPCR was 2 min at 95 °C, followed by 40 cycles of 15 s at 95 °C and 34 s at 60 °C. The *MsActin* gene was used as a house-keeping gene in qPCRs. Reactions were performed in biological triplicates and the data were analyzed using 2^−^^ΔΔCT^ method. The results were presented as means ± standard deviation (SD). Statistically significant differences (* for *p* < 0.05, or ** for *p* < 0.01) were calculated based on Student’s *t*-tests, and expression levels at each time point were all compared with 0 h for either the NaCl or PEG treatment. The relative expression level at 0 h was set as a value of 1 for each gene under stress treatment, and the relative expression level in the roots was set as value of 1 for different tissue samples. The primer sequences used in this study are listed in Table S3.

## Figures and Tables

**Table 1:** Properties of the predicted AQP proteins in *M. sativa*.

Number	TIGR-Locus	Gene Name	Homologous Gene	Locus	Aa	Predicted MW	Theoretical pI	Possible Localization
1	MsG0180005209.01.T01	*MsPIP1* *(MsPIP2;2)*	*MtPIP5*	Chr1: 88571578–88578205	288	30579.52	7.74	Cell membrane
2	MsG0380015447.01.T01	*MsPIP2*	*MtPIP3*	Chr3: 70296270–70298682	291	31059.17	8.90	Cell membrane
3	MsG0380018055.01.T01	*MsPIP3*	*MtPIP4*	Chr3: 103800315–103801076	254	26902.08	8.31	Cell membrane
4	MsG0480018608.01.T01	*MsPIP4*	*MtPIP7*	Chr4: 7319525–7323333	290	31159.12	8.82	Cell membrane
5	MsG0480020797.01.T01	*MsPIP5*	*MtPIP5*	Chr4: 48493988–48495990	248	26097.25	8.18	Cell membrane
6	MsG0480023653.01.T01	*MsPIP6*	*MtPIP10*	Chr4: 88108160–88113381	355	40069.02	8.75	Cell membrane
7	MsG0580024388.01.T01	*MsPIP7*	*MtPIP4*	Chr5: 4139613–4141330	197	21263.97	8.43	Cell membrane
8	MsG0580029158.01.T01	*MsPIP8*	*MtPIP8*	Chr5: 93033668–93036457	290	30906.21	8.61	Cell membrane
9	MsG0780040928.01.T01	*MsPIP9*	*MtPIP9*	Chr7: 84352446–84355540	288	30559.85	8.60	Cell membrane
10	MsG0180000870.01.T01	*MsTIP1*	*MtTIP2*	Chr1: 12403720–12404130	137	14437.45	5.66	Vacuole
11	MsG0180000871.01.T01	*MsTIP2*	*MtTIP1*	Chr1: 12412319–12413796	248	25781.49	5.64	Vacuole
12	MsG0180002324.01.T01	*MsTIP3*	*MtTIP2*	Chr1: 36718462–36720058	199	21587.50	7.04	Cell membrane
13	MsG0180002325.01.T01	*MsTIP4*	*MtTIP1*	Chr1: 36733969–36736328	282	29952.42	5.82	Vacuole
14	MsG0180004225.01.T01	*MsTIP5*	*MtTIP3*	Chr1: 75118675–75120930	177	18718.89	9.51	Vacuole
15	MsG0180004352.01.T01	*MsTIP6*	*MtTIP3*	Chr1: 76796413–76801040	415	45354.72	9.38	Vacuole
16	MsG0180006158.01.T01	*MsTIP7*	*MtTIP4*	Chr1: 100812909–100818492	583	64517.53	6.37	Vacuole
17	MsG0280011277.01.T01	*MsTIP8*	*MtTIP6*	Chr2: 81845004–81860681	810	87446.92	5.34	Vacuole
18	MsG0480019955.01.T01	*MsTIP9*	*MtTIP5*	Chr4: 30649828–30651833	255	26514.82	7.81	Cell membrane, Vacuole
19	MsG0480020913.01.T01	*MsTIP10*	*MtTIP7*	Chr4: 50479435–50486783	314	33087.29	8.89	Mitochondrion, Vacuole
20	MsG0480021749.01.T01	*MsTIP11*	*MtTIP4*	Chr4: 63840282–63843939	187	20199.99	7.59	Cell membrane
21	MsG0580024535.01.T01	*MsTIP12*	*MtTIP10*	Chr5: 6118582–6120774	250	25198.30	4.80	Vacuole
22	MsG0580027125.01.T01	*MsTIP13*	*MtTIP11*	Chr5: 54044943–54046569	206	21848.30	6.17	Cell membrane
23	MsG0680034456.01.T01	*MsTIP14*	*MtTIP11*	Chr6: 87915432–87916953	256	27105.24	6.79	Vacuole
24	MsG0780036905.01.T01	*MsTIP15*	*MtTIP12*	Chr7: 16035382–16038095	253	25952.35	5.30	Vacuole
25	MsG0780041015.01.T01	*MsTIP16*	*MtTIP13*	Chr7: 85529113–85530568	251	25377.42	5.60	Vacuole
26	MsG0880042325.01.T01	*MsTIP17*	*MtTIP14*	Chr8: 7123675–7134896	633	68553.42	5.94	Vacuole
27	MsG0180005353.01.T01	*MsNIP1*	*MtNIP2*	Chr1: 90755063–90759277	277	28709.35	7.70	Cell membrane
28	MsG0280006945.01.T01	*MsNIP2*	*MtNIP6*	Chr2: 8392964–8395613	176	18686.74	6.05	Cell membrane
29	MsG0280011459.01.T01	*MsNIP3*	*MtNIP8*	Chr2: 84287469–84289063	298	31898.00	8.64	Cell membrane
30	MsG0280011463.01.T01	*MsNIP4*	*MtNIP8*	Chr2: 84353257–84354088	177	18581.69	9.15	Cell membrane
31	MsG0380011693.01.T01	*MsNIP5*	*MtNIP9*	Chr3: 3582941–3583666	242	25411.55	5.88	Cell membrane, Vacuole
32	MsG0480018161.01.T01	*MsNIP6*	*MtNIP11*	Chr4: 1050114–1054907	306	31590.62	8.32	Cell membrane
33	MsG0480022137.01.T01	*MsNIP7*	*MtNIP12*	Chr4: 68715281–68716798	268	28455.95	6.30	Cell membrane
34	MsG0580024652.01.T01	*MsNIP8*	*MtNIP6*	Chr5: 7739808–7746624	424	45190.81	8.96	Cell membrane
35	MsG0580024653.01.T01	*MsNIP9*	*MtNIP3*	Chr5: 7756717–7759621	274	29033.09	7.77	Cell membrane
36	MsG0580027846.01.T01	*MsNIP10*	*MtNIP13*	Chr5: 69497858–69498560	131	14025.89	9.78	Cell membrane
37	MsG0580028354.01.T01	*MsNIP11*	*MtNIP13*	Chr5: 78973396–78975793	165	17535.42	6.18	Cell membrane
38	MsG0580028987.01.T01	*MsNIP12*	*MtNIP14*	Chr5: 89952725–89954888	267	28363.65	5.69	Cell membrane
39	MsG0780036257.01.T01	*MsNIP13*	*MtNIP3*	Chr7: 5446318–5449727	247	27704.80	4.95	Cell membrane
40	MsG0780036261.01.T01	*MsNIP14*	*MtNIP15*	Chr7: 5540627–5545064	275	28728.00	9.05	Cell membrane
41	MsG0680031712.01.T01	*MsSIP1*	*MtSIP2*	Chr6: 25135696–25141072	319	35007.47	9.38	Cell membrane
42	MsG0780040029.01.T01	*MsSIP2*	*MtSIP3*	Chr7: 72189472–72191112	199	20821.91	8.98	Cell membrane, Endoplasmic reticulum
43	MsG0780040134.01.T01	*MsSIP3*	*MtSIP3*	Chr7: 73492814–73494318	152	16306.22	7.70	Cell membrane

## Data Availability

All data in the present study are available in the public database as referred in the Material and Method part.

## Supplementary Materials

The following are available online at https://www.mdpi.com/article/10.3390/ijms23063342/s1.
